# Discovery of Cofactor Competitive Inhibitors against the Human Methyltransferase Fibrillarin

**DOI:** 10.3390/ph15010026

**Published:** 2021-12-24

**Authors:** Yun Shi, Ibrahim M. El-Deeb, Veronika Masic, Lauren Hartley-Tassell, Andrea Maggioni, Mark von Itzstein, Thomas Ve

**Affiliations:** Institute for Glycomics, Griffith University, Southport, QLD 4222, Australia; y.shi@griffith.edu.au (Y.S.); i.el-deeb@griffith.edu.au (I.M.E.-D.); v.masic@griffith.edu.au (V.M.); lauren.hartley@griffith.edu.au (L.H.-T.); a.maggioni@griffith.edu.au (A.M.)

**Keywords:** methyltransferase, S-adenosyl methionine, cofactor, fragment screening, inhibitors

## Abstract

Fibrillarin (FBL) is an essential and evolutionarily highly conserved S-adenosyl methionine (SAM) dependent methyltransferase. It is the catalytic component of a multiprotein complex that facilitates 2′-*O*-methylation of ribosomal RNAs (rRNAs), a modification essential for accurate and efficient protein synthesis in eukaryotic cells. It was recently established that human FBL (hFBL) is critical for Nipah, Hendra, and respiratory syncytial virus infections. In addition, overexpression of hFBL contributes towards tumorgenesis and is associated with poor survival in patients with breast cancer, suggesting that hFBL is a potential target for the development of both antiviral and anticancer drugs. An attractive strategy to target cofactor-dependent enzymes is the selective inhibition of cofactor binding, which has been successful for the development of inhibitors against several protein methyltransferases including PRMT5, DOT1L, and EZH2. In this work, we solved crystal structures of the methyltransferase domain of hFBL in apo form and in complex with the cofactor SAM. Screening of a fluorinated fragment library, via X-ray crystallography and 19F NMR spectroscopy, yielded seven hit compounds that competed with cofactor binding, two of which resulted in co-crystal structures. One of these structures revealed unexpected conformational variability in the cofactor binding site, which allows it to accommodate a compound significantly different from SAM. Our structural data provide critical information for the design of selective cofactor competitive inhibitors targeting hFBL, and preliminary elaboration of hit compounds has led to additional cofactor site binders.

## 1. Introduction

Fibrillarin (FBL) is an essential and highly conserved nucleolar localised protein [1] with roles in ribosome biogenesis and epigenetic regulation of ribosomal genes. In eukaryotes, FBL can be detected in the transition zone between the fibrillar center (FC) and dense fibrillar component (DFC), where ribosomal DNA (rDNA) transcription occurs, and in the DFC, where the pre-ribosomal RNA (rRNA) processing takes place [2,3]. Fibrillarin consists of an N-terminal glycine–arginine-rich domain (GAR), which is believed to be important for organisation of the nucleolus [4], and a C-terminal domain with a Rossmann fold that is characteristic of class I *S*-adenosylmethionine (SAM)-dependent methyltransferases [5]. In eukaryotes, FBL is the catalytic component of box C/D small nucleolar RNA–protein complexes (snoRNPs), which are responsible for 2′-*O*-methylation of ribose targets in rRNA [6]. 2′-*O*-methylation of rRNA has been reported as regulating the translational activity of ribosomes in yeast and humans, and to be crucial for proper development in mice [1,7,8]. FBL also participates in pre-rRNA cleavage by association with C/D box snoRNAs such as U3 or U14 [9], and regulates RNA polymerase I activity on rDNA gene promoters by methylating glutamine 104 of the histone H2A tail via an unknown mechanism [10].

Several studies have highlighted the overexpression of human FBL (hFBL) in cancer, including prostatic neoplasia [11], breast cancer [12,13], and hepatocellular carcinoma [14]. High expression levels of hFBL are accompanied by rRNA methylation pattern alterations, decreased translational efficiency, and an increase of internal ribosome entry site (IRES)-dependent translation initiation of key cancer genes [12]. As a result, hFBL has been proposed as a therapeutic target that could lower the genotoxic effects of anti-cancer treatment [15].

A biosafety level 4 genome-wide siRNA screen also revealed that hFBL is required for Nipah and Hendra virus infections [16]. These viruses are highly lethal (risk group 4) zoonotic paramyxoviruses causing severe, rapidly progressive encephalitis in humans [17]. The methyltransferase activity of hFBL was demonstrated to be required for viral RNA and protein syntheses, indicating a crucial role for hFBL in the RNA replication phase of infection. Infections by other paramyxoviruses, including respiratory syncytial virus, were also dependent on hFBL expression [16], suggesting that this enzyme can be targeted therapeutically to combat a range of paramyxovirus infections.

Methyltransferases have emerged as an attractive drug target class in oncology and other disease areas, and inhibitors targeting both DNA and protein methyltransferase have been reported. Inhibitors directly competing with the cofactor SAM have been developed for several protein methyltransferases including the arginine methyltransferase PRMT5, the histone lysine methyltransferase DOTL1 and EZH2, the catalytic subunit of Polycomb repressive complex 2, and are currently in various phases of clinical trial [18]. However, there are no inhibitors or chemical probes reported that specifically target the 2′-*O*-ribose methyltransferase function of hFBL.

One increasingly important strategy used to identify small molecule binders is fragment-based drug discovery (FBDD) [19,20,21]. FBDD employs fragment-sized compounds that usually comply with the ‘rule of three′ [22] for the initial screening against a biomolecular target. In comparison to traditional high-throughput screening, which use drug-like compounds adhering to the ‘rule of five′ [23], FBDD has better chances of hit identification thanks to its more efficient sampling of the chemical space [24]. In addition, the smaller sized fragment hits also have higher ligand efficiency [25] and amenability to structural optimisation [26]. FBDD has already been utilised to identify binders and develop potent inhibitors of methyltransferases [27,28,29].

In order to identify novel chemicals that bind to and inhibit SAM binding to hFBL, we determined crystal structures of the methyltransferase domain in both apo and cofactor bound states, and carried out crystallography and nuclear magnetic resonance (NMR) spectroscopy-based fragment screening campaigns using a library of 388 fluorinated fragment compounds. Two types of fragments were identified that bind to the cofactor site pocket and compete with SAM for binding to hFBL. One of the fragments revealed conformational variability in the cofactor binding site, which allows it to accommodate a compound significantly different from SAM. These high-resolution crystal structures of hFBL provide great insight into its binding with small molecules, paving the way for structure-based design and elaboration of inhibitors that can serve as chemical probes for its potential as an anti-cancer and/or anti-viral target.

## 2. Results

### 2.1. Apo and SAM-Bound hFBL Structures

**Figure 1:**
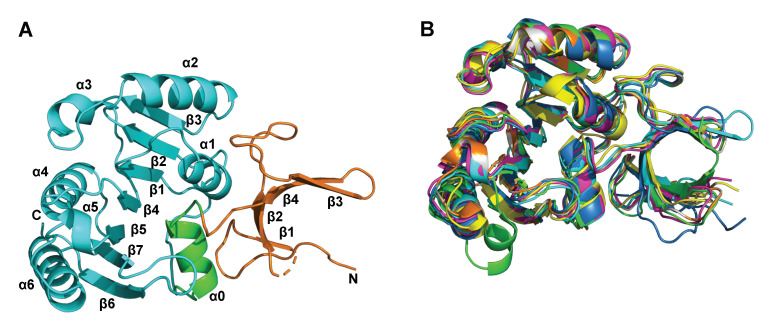
hFBL crystal structure. (**A**) Structure of hFBL (cartoon representation). The crystal structure consists of 11 β-strands and 7 α-helices that fold into an N-terminal (orange) and a C-terminal (cyan) domain and a short connecting helix (a0, green). (**B**) Superposition of representative FBL orthologs from *Aeropyrum pernix* (green, PDB ID 4DF3), *Methanocaldococcus jannaschii* (blue, PDB ID 1G8S), *Pyrococcus furiosus* (purple, PDB ID 3NMU), *Saccharolobus solfataricus* (orange, PDB ID 3PLA), *Chaetomium thermophilum* (grey, PDB ID 5JPQ), and *Saccharomyces cerevisiae* (yellow, PDB ID 6ND4) are shown.

**Figure 2:**
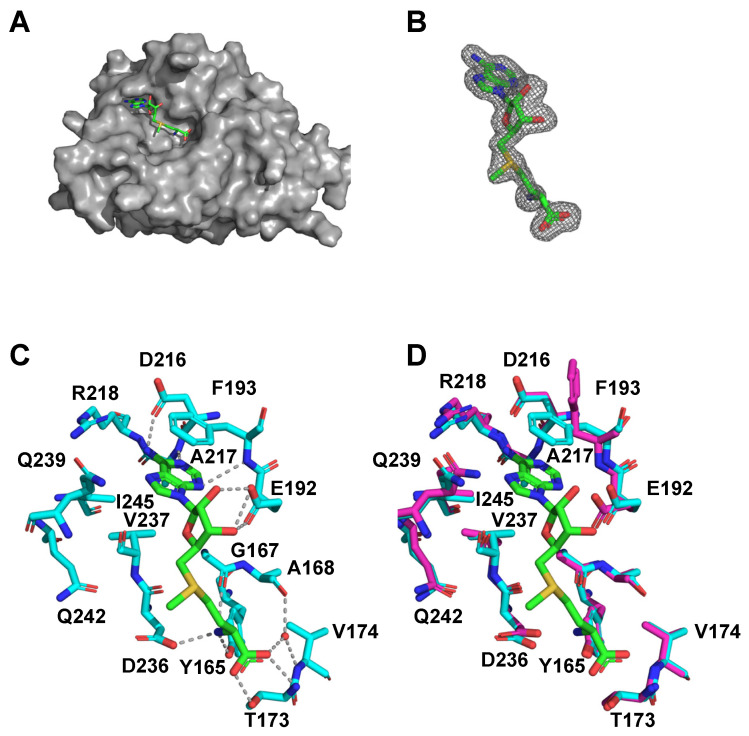
hFBL^SAM^ crystal structure. (**A**) Surface representation (grey) of the hFBL^SAM^ complex. SAM is shown in stick representation (green). (**B**) Standard omit *mFo-DFc* map (grey mesh, contoured at 3.0 σ) of the SAM molecule. (**C**) Stick representation of SAM (green) and residues in the cofactor binding pocket of hFBL (cyan). (**D**) Superimposition of the hFBL^SAM^ (cyan) and hFBL^Apo^ (purple) structures. Oxygen, nitrogen and sulphur atoms are coloured in red, blue, and orange, respectively.

We determined crystal structures of the catalytic domain (residues 85–321) of human fibrillarin (hFBL) in apo (hFBL^Apo^; PDB code: 7SE6) and SAM-bound (hFBL^SAM^; PDB code: 7SE7) states to 1.99 and 1.75 Angstrom resolutions, respectively (Figure 1A and Figure 2 and Table S1). The hFBL^Apo^ and hFBL^SAM^ structures are almost identical with an overall RMSD value of 0.2 for 229 Cα atoms, and the structures are superimposable with that of fibrillarin homolog structures from both archaeal and fungal species (Figure 1B and Table S2). SAM is bound in a conserved pocket in the C-terminal region of the protein, which adopts the typical SAM-dependent methyltransferases fold, consisting of a seven-stranded β-sheet (β1–7) sandwiched by six α-helices (α1–6) [5]. The binding mode of SAM is very similar to structures of archaeal fibrillarin homologs in complex with SAM and S-Adenosyl-l-homocysteine (SAH) [30,31] and hFBL in complex with methylthioadenosine (MTA) (Supplemental Figure S1A–C). Only minor changes are observed in hFBL^SAM^ compared to hFBL^Apo^ upon SAM binding (Figure 2C,D). The aromatic sidechain of F192 undergoes a significant rotation upon stacking with the adenine moiety of SAM. In addition, subtle movements in the sidechains of E191, D236, Q239, and R218 are observed upon cofactor binding.

### 2.2. Characterisation of hFBL–SAM Interactions

The interaction between hFBL and SAM was studied using isothermal titration calorimetry (ITC), surface plasmon resonance (SPR), and saturation transfer difference (STD) NMR. As predicted from the crystal structure, ITC experiments showed a single site binding model for SAM (Supplemental Figure S1D). The thermodynamic binding parameters (the change in enthalpy Δ*H*, the number of binding sites *N*, and the dissociation constant *K*_d_) were calculated from ITC data fitting as follows: Δ*H* = −31.56 ± 4.67 kJ/mol, *N* = 0.909 ± 0.005, *K*_d_ = 14 ± 0.8 µM. The dissociation constant for the hFBL–SAM interaction determined by SPR was higher but within the same order of magnitude (*K*_d_ = 33.7 ± 7.7 µM). The binding affinity is similar to the reported *K*_d_ value for the *Archaeoglobus fulgidus* FBL–SAM interaction (10.5 ± 1 µM) [32]. STD NMR assays also confirmed SAM binding to hFBL in solution, with the aromatic resonances from the adenine moiety of SAM showing good STD NMR signals (Supplemental Figure S2).

MD simulations of both hFBL^Apo^ and hFBL^SAM^ were conducted to further examine SAM interactions with hFBL. While SAM binding showed an overall stabilising effect on hFBL structure, it appeared to destabilise the loop W137-K143, with the binding site residue R141 being the most notable (Supplemental Figure S3).

### 2.3. Fragment Screening

Fragment screening was carried out via both ^19^F NMR spectroscopy and X-ray crystallography using a library of 388 fluorinated compounds with great structural diversity (Figure S4). This library was originally assembled for ^19^F NMR detected screening, with the compounds grouped into 20 pools, each of which consists of up to 20 compounds. 

**Figure 3:**
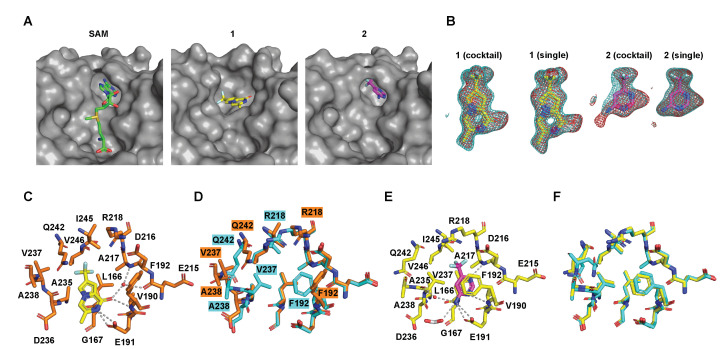
hFBL crystal structures with fragment hits. (**A**) Surface representation (grey) of the hFBL:SAM, hFBL:**1** and hFBL:**2** and complexes. The hFBL protein backbone is displayed in cartoon representation (**1**, orange; **2**, yellow; SAM, cyan) while the ligands are shown in stick representation (1, yellow; 2, magenta; SAM, green). Oxygen, nitrogen, sulphur, and fluorine atoms are coloured in red, blue, light orange, and pale cyan, respectively. (**B**) Standard omit mFo-DFc (red mesh, contoured at 3.0 σ) and polder (cyan mesh, contoured at 4.0 σ) maps of **1** and **2**. (**C**) Stick representation of **1** and residues in the cofactor binding pocket of hFBL. (**D**) Superimposition of the hFBL:SAM and hFBL:**1** structures. Residues undergoing extensive rearrangements are labelled. (**E**) Stick representation of **2** and residues in the cofactor binding pocket of hFBL (citrate, grey). (**F**) Superimposition of the hFBL:SAM and hFBL:**2** structures.

**Figure 4:**
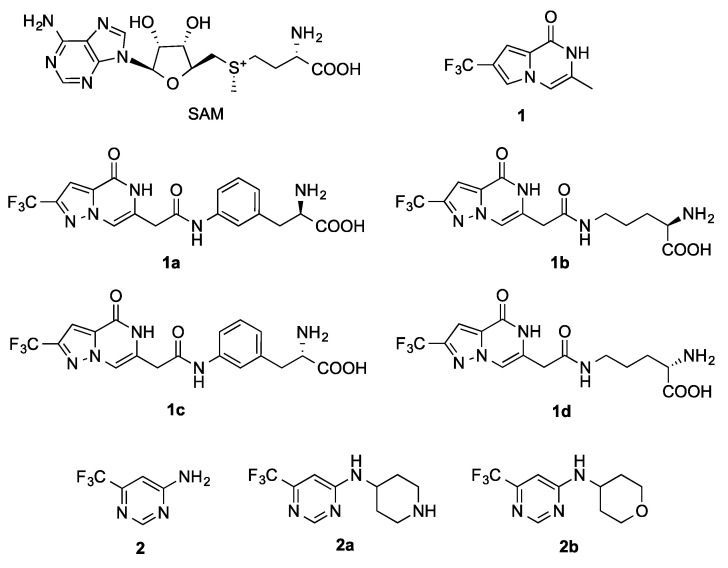
Chemical structures of ligands shown to bind at the cofactor site of hFBL.

**Figure 5:**
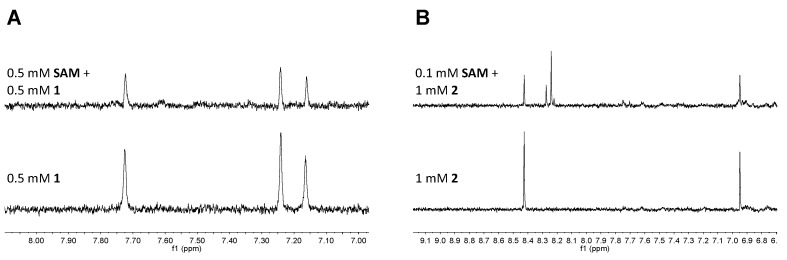
STD NMR spectra showing competitive binding between cofactor SAM and fragment hits **1** and **2**, respectively, with hFBL. (**A**) STD NMR spectra showing the binding of 0.5 mM **1** to 35 µM hFBL in the absence (bottom expansion) and presence (top expansion) of 0.5 mM SAM, with 1024 scans and 2 s saturation time. (**B**) STD NMR spectra showing the binding of 1 mM **2** to 40 µM hFBL in the absence (bottom expansion) and presence (top expansion) of 0.1 mM SAM, with 512 scans and 3 s saturation time.

The X-ray crystallography screen resulted in two fragments (Figure 3A,B), PS-6655 (**1**), and FS-2818 (**2**) (Figure 4), that could be unambiguously identified from the Fo–Fc difference electron density maps at 1.75 Å (PDB code: 7SE8) and 1.91 Å resolution (PDB code: 7SEA), respectively (Figure 3 and Supplemental Table S1). To confirm these results, hFBL crystals were soaked with compounds **1** and **2**, individually, and X-ray diffraction datasets were collected at 1.75 Å and 1.81 Å resolution for fragment **1** (PDB code: 7SE9) and **2** (PDB code: 7SEB)**,** respectively (Supplemental Table S1). In the single component soaks, both fragments bound to the active site region in the same manner as was observed in the multi-component soaks (Figure 3B). To investigate if these two fragments can compete with SAM binding in solution, STD NMR assays were performed. The presence of SAM led to decreases in the STD NMR signals of both fragment hits, confirming their competition with cofactor binding to hFBL (Figure 5).

In ^19^F NMR detected screening, 9 fragment hits were identified. STD NMR competition assays revealed that five of these compounds are SAM competitive binders, as their STD NMR signals decreased in the presence of SAM (Supplemental Figure S5). However, none of these NMR hits could be detected by X-ray crystallography when soaked individually with hFBL crystals.

### 2.4. Details of the Binding Modes of Compounds **1** and **2**

Compound **1** binds to the adenosine region of the hFBL cofactor binding site by an induced fit mechanism, which involves a significant rearrangement in the loop connecting the β4 strand and α5 helix (Figure 3A,C,D). The compound induces a significant shift of the protein backbone around D236-Q242 resulting in extensive movement of the side chains of V237 (5.9 Å) and Q242 (5.2 Å). The movement of the side chain of V237, which forms a hydrophobic interaction with the adenine moiety of SAM in hFBL^SAM^, results in displacement of the side chain of Q242 from a buried to a solvent exposed position, and exposure of a new hydrophobic pocket (L166, A235, V237, I245, and V246) that accommodates the trifluoromethyl group of the compound (Figure 3A,C,D). Compound **1** also forms hydrogen bonds with the side chain carboxylate of E191, the backbone nitrogen of F192, and a water molecule that interacts with the backbone nitrogen of A217 and the backbone carbonyl of E216.

Compound **2** does not induce rearrangements of the active site region of hFBL. Instead, it mimics the binding mode of the adenosine moiety of SAM in hFBL^SAM^ with the pyrimidine ring forming a π stack interaction with the aromatic side chain of F192 (Figure 3A,E,F). It also forms hydrogen bonds with the side chain carboxylate of E191, the backbone nitrogens of F192 and A217, and a formic acid molecule.

### 2.5. Preliminary Elaboration of Fragment Hits

In order to generate better cofactor competitive binders with additional interactions and possibly improved affinities, we set out to elaborate verified fragment binders into compounds of larger sizes. Based on the bound structures of **1**, **2**, and SAM, we designed derivatives of **1** and **2** with the consideration that these derivatives could be synthesised in-house and obtained from commercial sources, respectively, and subjected them to molecular docking. We selected 4 derivatives of **1**, namely **1a**, **1b**, **1c**, and **1d** (Figure 4). They all have an amide linker that connects a synthetically convenient analogue of **1** and an amino acid, the latter of which showed potential to stabilise the loop W137-K143 via ionic interactions between their terminal carboxylate group and the amino moiety on the side chain of K143 (Figure S6A). We also selected two derivatives of **2**, namely **2a** and **2b** (Figure 4), as their docked poses were in a similar position to that of **2** (Figure S6B) with better docking scores (Supplemental Table S2).

**Scheme 1:**
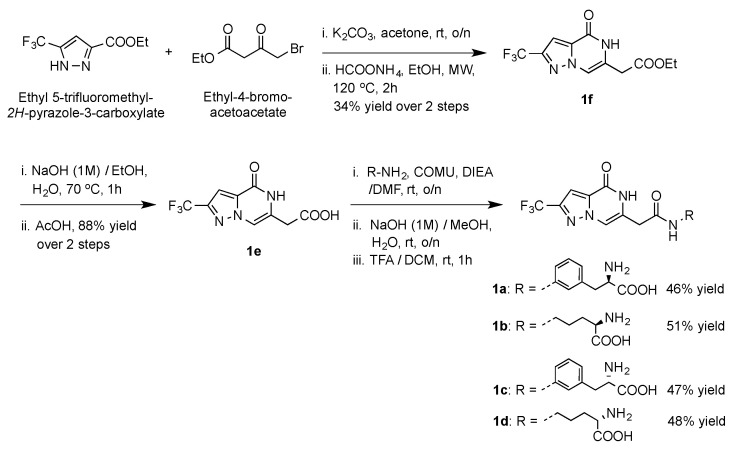
Synthesis of compounds **1a**–**d**.

The synthesis of compounds **1a**–**d** (Scheme 1) started with the coupling of ethyl 5-trifluoromethyl-*2H*-pyrazole-3-carboxylate and ethyl-4-bromo-acetoacetate in the presence of potassium carbonate in acetone, followed by cyclisation under microwave (MW) irradiation in ethanol, and in the presence of ammonium formate, to yield intermediate **1f** in 34% yield over two steps. Base-catalysed deprotection of the ester group of **1f** with aqueous NaOH yielded the intermediate **1e** in 88% yield. (1-Cyano-2-ethoxy-2-oxoethylidenaminooxy)dimethylamino-morpholino-carbenium (COMU) catalysed amide coupling of intermediate **1e** with properly protected amino acids in dimethylformamide (DMF) yielded the protected amides. These amino acids were commercially sourced to ensure enantiomeric identity and purity. Base-catalysed deprotection of the ester group with aqueous NaOH followed by BOC-deprotection with anhydrous TFA yielded the final products **1a**–**d** in the yields shown in Scheme 1.

**Figure 6:**
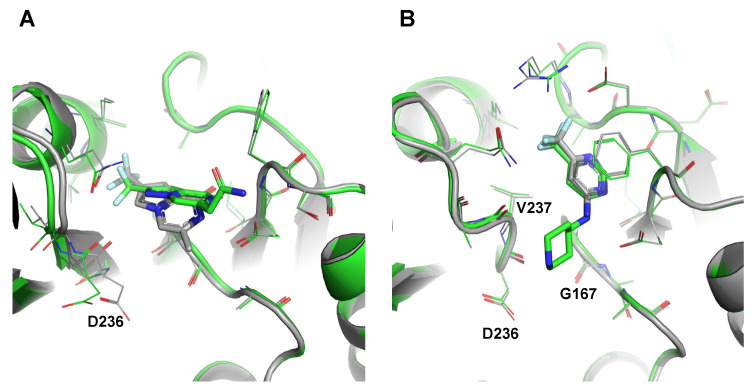
hFBL crystal structures with fragment derivatives. (**A**) Superimposition of the hFBL:**1** (grey) and hFBL:**1a** (green) complexes. (**B**) Superimposition of the hFBL:**2** (grey) and hFBL:**2a** (green) complexes. Ligand and interacting residues are displayed in thick and thin stick representation, respectively (oxygen, red; nitrogen, blue; fluorine, pale cyan). The protein backbone is displayed in cartoon representation.

All derivatives were subjected to crystallographic soaking in a similar fashion to that of compounds **1** and **2** and complex structures were obtained for **1a** (PDB code: 7SEC) and **2a** (PDB code: 7SED) (Figure 6, Figure S6C, and Supplemental Table S1).

A different orientation of the fused bicyclic ring was observed for **1a** from **1** with concomitant movements of some binding site residues, especially D236 (Figure 6A). In addition, electron density for **1a** was not observed beyond the amide linker, which appeared to be turning away from the catalytic site and pointing towards the bulk solvent. This indicates that the extended D-phenylalanine moiety did not bind as well as the methionine portion of SAM, and was consequently exposed to the bulk solvent, which in turn affects the orientation of the fused bicyclic ring. Indeed, the binding affinity of **1a** turned out to be similar to that of **1** (Supplemental Table S3). In addition to **1a**, **1c**, **1d**, and a synthetic intermediate without the amino acid **1e** were also shown to compete with SAM binding to hFBL in solution by STD NMR assays (Supplemental Figure S7A,B). However, **1b**, **1c**, and **1d** were not able to be soaked into the binding site of hFBL, probably due to unfavourable interactions from their extended phenylalanine or norvaline moiety and their weaker binding affinities (Supplemental Table S3).

By contrast, **2a** showed very similar binding modes to those predicted by molecular docking, with its aromatic pyrimidine ring sitting close to that of **2,** and maintained all interactions observed for **2** (Figure 6B). Additionally, the extended piperidine ring in **2a** was engaged in multiple hydrophobic interactions with residues G167, D236, and V237 with a stable chair conformation. Notably, the binding affinity of **2a** improved by two orders of magnitude from **2** (Supplemental Table S2). Further, both **2a** and **2b** were shown to compete with cofactor binding by STD NMR assays (Supplemental Figure S7C).

## 3. Discussion

The 2′-*O*-methyltransferase function of hFBL is a potential therapeutic target in both cancers and viral infections, but chemical probes that can explore and/or validate this potential are currently lacking. FBL cannot bind to the substrate, rRNA, by itself and additional proteins in the snoRNP complex are responsible for binding RNA (SNU13) and bringing the RNA and the FBL cofactor binding site into close proximity (NOP56 and NOP58) [33]. Hence, only the cofactor binding site is a candidate for hFBL inhibitor development. In the present study, we determined, to our knowledge, the first crystal structures of hFBL in ligand-free state, bound to the native cofactor SAM, and in complex with two fragments that can compete with SAM for binding to the cofactor site. One of these fragment complexes (compound **1**) revealed structural plasticity at the cofactor site as binding of the fragment lead to exposure of a hydrophobic cavity that accommodated the tri-fluoro moiety of the compound. The hydrophilic nature of the cofactor binding site in SAM dependent methyltransferases has in general been challenging for drug discovery against this class of proteins, and exploitation of unique hydrophobic cavities has been critical for developing DOTL1 and EZH2 cofactor competitive inhibitors that are sufficiently polar to exploit the cofactor binding site, yet sufficiently hydrophobic to cross the membrane [34,35,36,37]. Our discovery of conformational dynamics and a hydrophobic cavity at the hFBL cofactor site will help to facilitate structure-guided design of hFBL inhibitors. Indeed, our preliminary elaboration of fragment hits provided proof of concept for an FBDD approach, as growing fragments were shown to maintain binding interactions at the cofactor site. Although the improvement in binding affinity was not particularly substantial in this work, future studies could explore different derivatives of **1** to better mimic the interactions from the methionine portion of SAM and further extensions to **2a** to engage in additional and stronger interactions with the cofactor binding site.

Our fragment library was originally built for convenient application of ^19^F NMR detected screening, with the separation of ^19^F NMR resonances being the most important criterium for the construction of pools for fast screening. However, the robust crystallisation system we established for hFBL allowed us to apply exactly the same set of pools for crystallographic screening, and bound fragments were unambiguously identified from the electron density map. To our knowledge, such a large number of fragments per pool (20 compounds) is unprecedented for crystallographic screening [38]. In addition, we observed no overlap between hits identified by NMR screening and by crystallographic screening. This is not surprising, as different screening methods are expected to yield different results [39], and the overlap between NMR and X-ray hits was shown to be small for another fragment library of similar size [40]. Hence, it is advantageous to employ two or multiple assays for fragment screening when a larger number of hits are desired.

## 4. Materials and Methods

### 4.1. Protein Production and Purification

Codon optimised cDNA of the methyltransferase domain of hFBL (residues 83–321, hFBL) was amplified by PCR and cloned into the pMCSG7 expression vector by ligation-independent cloning [41]. The resulting construct, which has an N-terminal TEV cleavable 6xHis-tag, was produced in *E. coli* BL21 (DE3) cells using the autoinduction method [42]. Cells were grown at 310 K until the mid-exponential phase (OD_600nm_ of 0.6–0.8) was reached. The temperature was then reduced to 293 K and the cultures were grown for approximately 16 h before harvesting. The cells were resuspended in lysis buffer (50 mM HEPES pH 7.5, 500 mM NaCl, 5% glycerol, and 0.5 mM TCEP) and lysed using sonication. The resulting lysate was clarified by centrifugation and the supernatant was supplemented with 30 mM imidazole and applied onto a 5 mL HisTrap FF column (GE Healthcare) preequilibrated with wash buffer (50 mM HEPES pH 7.5, 500 mM NaCl, 5% glycerol, 30 mM imidazole, and 0.5 mM TCEP). The column was then washed with 20 column volumes of the wash buffer and the bound protein was eluted using the elution buffer (50 mM HEPES pH 7.5, 500 mM NaCl, 5% glycerol, 250 mM imidazole, and 0.5 mM TCEP). The elution fractions were analysed by SDS-PAGE and the fractions containing hFBL were pooled, supplemented with TEV protease to remove the 6xHis-tag, and dialysed into the gel filtration buffer (10 mM HEPES pH 7.5, 500 mM NaCl, 10% glycerol, and 0.5 mM TCEP). After overnight TEV cleavage the sample was re-run over a HisTrap column preequilibrated in gel filtration buffer to remove the protease. Column flowthrough fractions containing cleaved protein were pooled together, concentrated, and further purified by gel filtration on a Superdex 75 HiLoad 26/60 column (GE Healthcare) pre-equilibrated with gel filtration buffer. The peak fractions were analysed by SDS-PAGE, and the fractions containing hFBL were pooled and concentrated to a final concentration of 16–20 mg/mL, and stored at −80 °C in 25 μL aliquots.

### 4.2. Fragment Library

A library of 388 fragments was assembled in-house using commercially sourced fluorinated compounds that comply with the ‘rule of three’ [22]. They were selected after quality checks that removed compounds with poor solubility, aggregation, and/or problematic ^19^F NMR resonances. The diversity of these 388 fluorinated fragments were evaluated by similarity scores calculated with a previously established protocol [43]. They were grouped in pools of 17 to 20 compounds, ensuring sufficient separation of ^19^F NMR resonances among all compounds of the same pool. This resulted in 20 pools of stock solutions in DMSO-d_6_, with the concentration of each fragment being 12.5 mM (mono- or di-fluorinated compounds) or 6.25 mM (tri-fluorinated compounds). These pools were subsequently subjected to screening with hFBL by X-ray crystallography and ^19^F NMR spectroscopy.

### 4.3. Crystallisation

Crystals of hFBL were grown by the hanging drop vapour diffusion method at 293 K with drops containing 1 μL of protein (16–20 mg/mL), and 1 μL of reservoir solution (2.75–3.3 M sodium formate) and appeared within 2–4 days. Consistency of crystal size and quality was greatly improved by microseeding.

### 4.4. Crystallographic Fragment Screening

Crystallographic fragment screening was carried out using the aforementioned pools of fluorinated fragments. Soaking drops were prepared by mixing 2 μL of each pool with 2 μL reservoir solution, leading to a final concentration of 6.25 mM or 3.125 mM for each fragment (50% (*v*/*v*) DMSO). HFBL crystals were then transferred to the soaking drop and soaked for a period of 48 h.

### 4.5. Preparation of hFBL-Compound Crystals

SAM, single fragments, and fragment derivatives were soaked into hFBL crystals at a concentration of 10–20 mM for 24–48 h. Soaking drops were prepared by mixing 2 μL of compound solution with 2 μL reservoir solution, followed by transfer of hFBL crystals to the soaking drop.

### 4.6. Data Collection and Processing, Structure Determination, and Refinement

The crystals were cryoprotected in Paratone-N and flash cooled at 100 K. X-ray diffraction data were collected from single crystals on the MX1 and MX2 beamlines at the Australian Synchrotron, using a wavelength of 0.9537 Å. The datasets were processed using XDS and scaled using Aimless in the CCP4 suite [44]. The structures were solved by molecular replacement using Phaser [45] and the hFBL:methylthioadenosine complex structure (PDB ID 2IPX) as a template. The models were refined using Phenix [46], and structure validation was performed using MolProbity [47]. The final structures have been deposited in the PDB. Data processing and refinement statistics are given in Supplemental Table S1. In the final refined models, no electron density was observed for residues 99–103 and 318–321, suggesting that these regions have a disordered or flexible conformation in the crystals.

### 4.7. Structural Analyses

Structural analyses were carried out using PyMOL (Schrödinger, New York, NY, USA). Figures were generated using PyMOL.

### 4.8. Molecular Dynamics Simulations

The hFBL^Apo^ and hFBL^SAM^ complex structures were used as the starting points of molecular dynamics (MD) simulations. They were first processed by Maestro (Schrodinger LLC) to add protons. GROMACS [48,49] was employed for MD simulations. The AMBER99SB force field [50] was adopted to parameterise protein residues, while parameters for the cofactor SAM were taken from a previous study [51]. Each structure was placed in a dodecahedral box with a minimal distance of 1.4 nm between the solute and box edge, followed by solvation with TIP3P water molecules. Salt ions were then added to a concentration of 0.15 M to balance ionic charge in the system. Energy minimisations were carried out with steepest descent integrator and conjugate gradient algorithm sequentially to achieve a maximum force of less than 500 kJ mol^−1^ nm^−1^ on any atom. The Verlet cut-off scheme [52] was used to evaluate short-range, non-bonded interactions, with both van der Waals and electrostatic interactions truncated at 0.8 nm. Long-range electrostatic interactions were treated by the particle mesh Ewald (PME) method [53,54]. The temperature was maintained at 298 K using a velocity rescaling thermostat [55] with a coupling constant of 0.1 ps, while the pressure was maintained at 1.0 atm using a Berendsen barostat [56] with a coupling constant of 1 ps. Simulations were performed with a time step of 2 fs, and all bonds involving hydrogen atoms were constrained by a parallel linear constraint solver (P-LINCS) [57]. Each system was equilibrated under a constant volume (NVT) ensemble for 100 ps and a constant pressure (NPT) ensemble for 100 ps. A harmonic position restraint with a force constant of 1000 kJ mol^−1^ nm^−2^ was applied to all the heavy atoms of non-solvent molecules. After equilibration, production MD simulations were conducted for 100 ns for each system without any constraints. Three replicate MD runs were performed for each system by varying the random seed for initial velocity generation. Analyses of MD trajectories were performed by inbuilt programs of GROMACS 5 on the final 80 ns of each 100-ns run to allow for equilibrium.

### 4.9. NMR Fragment Screening

Each NMR sample (200 µL) was prepared in a solution consisting of 175 µL HBS buffer (0.01 M HEPES pH 7.5, 500 mM NaCl, 0.5 mM TCEP, and 1% glycerol), 20 µL D_2_O, and 5 µL DMSO-d_6_. The final protein concentration was 20 µM, while the final fragment concentration was 312.5 µM for mono- and di-fluorinated fragments and 156.25 µM for tri-fluorinated fragments. Each sample was submitted for ^19^F NMR experiment automatically by IconNMR, employing a customised pulse sequence that consisted of a spin echo sequence with a composite smoothed chirp 180 pulse [58]. A total of 400 scans were executed at 298 K for each experiment. Control samples without protein were also subjected to the same ^19^F NMR experiments. Screening hits were identified by comparison of ^19^F NMR spectra from protein present samples and that from protein absent samples, as a significant reduction of NMR peak intensity (height) indicates fragment binding to the protein.

### 4.10. Molecular Docking

To select fragment derivatives, molecular docking was performed by the Glide program (Schrödinger, New York, NY, USA). SMILES structures of 48 designed derivatives of **1** (including **1** itself) were processed by the LigPrep panel within Maestro 11 (Schrödinger, New York, NY, USA) with default parameters. Likewise, 25 commercially available derivatives of **2** (including **2** itself) were processed in the same way. The resulting three-dimensional structures of these compounds were used as ligands for molecular docking. Crystal structures of hFBL in complex with **1** and **2**, respectively, were processed by the Protein Preparation Wizard within Maestro 11 using default settings. All water molecules in the crystal structures were removed, and the processed protein structures were used as receptors for molecular docking. Grid boxes of the default size (30 × 30 × 30 Å) were generated with their centres on **1** and **2**, respectively. Standard precision docking was performed by Glide [59] for each set of derivatives with all other settings kept as default.

### 4.11. STD NMR

Each sample for STD NMR (200 µL) consisted of 175 µL HBS buffer, 20 µL D_2_O, and 5 µL DMSO-d_6_ with 20–40 µM hFBL and 0.5–1 mM ligand. Each sample was transferred into a 3 mm Bruker NMR tube rated for 600 MHz data acquisition, and spectra were acquired with a Bruker Avance 600 MHz NMR spectrometer equipped with ^1^H/^13^C/^15^N triple resonance cryoprobe at 298 K. The pulse sequence STDDIFFGP19.3, inbuilt within the Bruker TopSpin^TM^ program, was employed to acquire STD NMR spectra [60]. This pulse sequence consists of a 3–9–19 water suppression pulse, the parameters of which were obtained from optimisation of ^1^H watergate 3–9–19 suppression pulse (P3919GP) [61], to suppress resonance from H_2_O. The on-resonance irradiation was set close to protein resonances between −0.15 ppm and 0.77 ppm, whereas the off-resonance irradiation was set far away from any protein or ligand resonances at 300 ppm. A relaxation delay of 4 s was used, out of which a saturation time of 2 or 3 s was used to irradiate the protein with a train of 50 ms Gaussian shaped pulses. The number of scans were kept between 400 and 1024 depending on instrument availability.

### 4.12. Chemical Synthesis

*General information:* Reagents and dry solvents purchased from commercial sources were used without further purification. Anhydrous reactions were carried out under an atmosphere of argon, using oven-dried glassware. Reactions were monitored using thin layer chromatography (TLC) on aluminium plates precoated with silica gel 60 F254 (Merck, Melbourne, Australia). Developed plates were observed under UV light at 254 nm and then visualised after application of a solution of ninhydrin in EtOH (0.2% *w*/*v*) and heating. Flash chromatography was performed on silica gel 60 (0.040–0.063 mm) using distilled solvents. ^1^H and ^13^C NMR spectra were recorded at 400 and 100 MHz, respectively, on a Bruker Avance 400 MHz spectrometer. Chemical shifts (δ) are reported in parts per million (ppm), relative to the residual solvent peak as internal reference (DMSO: 2.50 (pent) for ^1^H, 39.51 (hept) for ^13^C; D_2_O: 4.79 (s) for ^1^H). 2D COSY and HSQC experiments were run to support assignments. Low resolution mass spectra (LRMS) were recorded in electrospray ionization mode on a Bruker Daltonics Esquire 3000 ESI spectrometer, using positive mode. The protection of the amino acids used for the amide coupling followed typical procedures to that mentioned in the literature [62,63].

*Ethyl 2-(4-oxo-2-(trifluoromethyl)-4,5-dihydropyrazolo[1,5-a]pyrazin-6-yl)acetate* (***3***)*,* Ethyl 4-bromo-acetoacetate (335 μL, 2.4 mmol) was added to a solution of ethyl 5-trifluoromethyl-*2H*-pyrazole-3-carboxylate (500 mg, 2.4 mmol) in acetone (10 mL), followed by finely powdered dry K_2_CO_3_ (400 mg, 12.9 mmol). The reaction mixture was stirred at room temperature (rt) overnight (o/n) and was then concentrated under reduced pressure to remove the reaction solvent. The crude product was diluted with dichloromethane (DCM) (100 mL) and washed with water (50 mL). The organic layer was separated, washed with brine, dried over Na_2_SO_4_, and concentrated under reduced pressure. The residue was dissolved in absolute EtOH (5 mL) followed by the addition of ammonium formate (2.27 g, 36 mmol). The mixture was heated under microwave irradiation at 393 K for 2 h. After cooling to rt, the solvent was removed, the residue was diluted with H_2_O (20 mL), and was extracted with EtOAc (50 mL × 2). The combined organic extract was washed with brine, dried over Na_2_SO_4_, and concentrated under reduced pressure. Purification of the residue by silica gel chromatography using hexane:EtOAc (3:2) yielded pure **3** (235 mg, 34% yield) as a white powder. ^1^H NMR (400 MHz, DMSO-*d_6_*): δ 1.21 (t, *J* = 7.0 Hz, 3H), 3.64 (s, 2H), 4.13 (q, *J* = 7.1 Hz, 2H), 7.50 (s, 1H), 7.79 (s, 1H), 11.70 (s, 1H); ^13^C NMR (101 MHz, DMSO-*d_6_*): δ 14.52, 35.23, 61.33, 103.65 (d, *J* = 2.3 Hz), 109.77, 121.50 (q, *J* = 268.7 Hz), 126.45, 134.10, 141.71 (q, *J* = 38.0 Hz), 155.34, 169.28; LRMS [C_11_H_10_F_3_N_3_O_3_] (*m*/*z*): (+ve ion mode) 311.9 [M+Na]^+^.
*2-(4-Oxo-2-(trifluoromethyl)-4,5-dihydropyrazolo[1,5-a]pyrazin-6-yl)acetic acid* (**4**), Compound **3** (200 mg, 0.69 mmol) was suspended in EtOH/H_2_O (1:1, 10 mL) and NaOH (1M) was added to the suspension (under stirring) in a dropwise manner until pH = 14. The reaction mixture was heated at 243 K °C for 1 h, then allowed to cool down to rt. The reaction mixture was then acidified to pH = 4 using AcOH, concentrated under reduced pressure, and the residue was purified by silica gel chromatography using EtOAc:MeOH:H_2_O (9:1:0.5) to yield the pure acid **4** (160 mg, 88% yield) as a pale yellow powder. ^1^H NMR (400 MHz, DMSO-*d_6_*): δ 3.54 (s, 2H), 7.49 (s, 1H), 7.78 (s, 1H), 11.68 (s, 1H); ^13^C NMR (101 MHz, DMSO-*d_6_*): δ 35.45, 103.55 (d, *J* = 2.6 Hz), 109.60, 121.52 (q, *J* = 268.7 Hz), 127.08, 134.04, 141.62 (q, *J* = 38.1 Hz), 155.35, 170.80; LRMS [C_9_H_6_F_3_N_3_O_3_] (*m*/*z*): (+ve ion mode) 283.1 [M+Na]^+^.
*General method for the synthesis of***1a**–**d**, To a solution of the carboxylic acid **4** (50 mg, 0.19 mmol) in anhydrous DMF (2 mL) under argon the amine component (0.38 mmol) and COMU (160 mg, 0.38 mmol) were added followed by *N*,*N*-Diisopropylethylamine (DIEA) (132 μL, 0.76 mmol). The reaction mixture was stirred at rt o/n and was then concentrated under reduced pressure. The residue was triturated with H_2_O (10 mL) and extracted with EtOAc (50 mL). The organic layer was washed with brine, dried over Na_2_SO_4_, and concentrated under reduced pressure to yield the crude product. Purification by silica gel chromatography using the proper solvent system yielded the pure amide. The protected amide was suspended in a MeOH:H_2_O mixture (1:1, 2 mL), treated with NaOH (1M) to pH = 14, stirred at rt o/n, then the reaction was acidified with Amberlite@IR-120 (H^+^) resin (to pH = 5), filtered, and washed with MeOH (10 mL) and H_2_O (10 mL). The combined filtrate and washings were then concentrated under vacuum and the residue was dissolved in DCM (2 mL) and treated with TFA (300 μL, 3.8 mmol, 20 eq). After stirring for 1 h at rt, the solvent and excess TFA were removed by concentration and reduced pressure, and the residue was purified by silica gel chromatography using the suitable solvent system to yield the pure, deprotected amide **1a**–**d**.
(R)-2-Amino-3-(3-(2-(4-oxo-2-(trifluoromethyl)-4,5-dihydropyrazolo[1,5-a]pyrazin-6-yl)acetamido)phenyl)propanoic acid IE1775-42 (**1a**), The amide was formed by coupling with (R)-methyl 3-(3-aminophenyl)-2-((tert-butoxycarbonyl)amino)propanoate and the protected product was purified by silica gel chromatography using Hexane:EtOAc:MeOH (4:2:0.5). The final deprotected amide (**1a**) was obtained in 46% yield (over three steps) after purification by silica gel chromatography using EtOAc:MeOH:H_2_O (9:2:1) as a solvent. ^1^H NMR (400 MHz, D_2_O): δ 2.81 (dd, *J* = 13.5, 7.5 Hz, 1H), 2.99 (dd, *J* = 13.5, 5.5 Hz, 1H), 3.49 (dd, *J* = 7.5, 5.5 Hz, 1H), 3.66 (d, *J* = 7.0 Hz, 1H), 7.10 (d, *J* = 6.3 Hz, 2H), 7.27 (s, 1H), 7.32–7.41 (m, 2H), 7.78 (s, 1H); ^13^C NMR (101 MHz, D_2_O): δ 23.24, 40.64, 57.34, 98.63 (d, *J* = 2.8 Hz), 109.98, 120.47, 121.21 (d, *J* = 268.3 Hz), 123.01, 126.62, 129.15, 132.44, 136.65, 136.76, 139.33, 140.87 (q, *J* = 38.0 Hz), 163.73, 171.83, 182.23; LRMS [C_18_H_16_F_3_N_5_O_4_] (*m*/*z*): (-ve ion mode) 421.9 [M-H]^−^.
*(R)-2-Amino-5-(2-(4-oxo-2-(trifluoromethyl)-4,5-dihydropyrazolo[1,5-a]pyrazin-6-yl)acetamido)pentanoic acid IE1775-41* (**1b**), The amide was formed by coupling with (*R*)-methyl 5-amino-2-((*tert*-butoxycarbonyl)amino)pentanoate and the protected product was purified by silica gel chromatography using Hexane:EtOAc:MeOH (4:2:0.5). The final deprotected amide (**1b**) was obtained in 51% yield (over three steps) after purification by silica gel chromatography using EtOAc:MeOH:H_2_O (7:2:1) as a solvent. ^1^H NMR (400 MHz, D_2_O): δ 1.49–1.68 (m, 4H), 3.24 (ddd, *J* = 9.4, 7.9, 4.5 Hz, 3H), 3.49 (s, 2H), 7.10 (s, 1H), 7.72 (s, 1H); ^13^C NMR (101 MHz, DMSO-*d_6_*): δ 24.76, 31.75, 39.44, 41.09, 55.59, 98.73, 109.84, 121.19 (d, *J* = 268.2 Hz), 132.41, 136.58, 140.91 (q, *J* = 38.4 Hz), 163.54, 172.87, 182.73; LRMS [C_14_H_16_F_3_N_5_O_4_] (*m*/*z*): (+ve ion mode) 398.1 [M+Na]^+^.
*(S)-2-Amino-3-(3-(2-(4-oxo-2-(trifluoromethyl)-4,5-dihydropyrazolo[1,5-a]pyrazin-6-yl)acetamido)phenyl)propanoic acid IE1775-50* (**1c**), The amide was formed by coupling with (*S*)-methyl 3-(3-aminophenyl)-2-((*tert*-butoxycarbonyl)amino)-propanoate and the protected product was purified by silica gel chromatography using Hexane:EtOAc:MeOH (4:2:0.5). The final deprotected amide (**1c**) was obtained in 47% yield (over three steps) after purification by silica gel chromatography using EtOAc:MeOH:H_2_O (9:2:1) as a solvent. ^1^H NMR (400 MHz, D_2_O): δ 1.92 (s, 1H), 2.87 (dd, *J* = 13.8, 7.7 Hz, 1H), 3.05 (dd, *J* = 13.8, 5.4 Hz, 1H), 3.61 (dd, *J* = 7.7, 5.4 Hz, 1H), 7.10 (m, 1H), 7.15 (s, 1H), 7.28 (s, 1H), 7.32–7.40 (m, 2H), 7.74 (s, 1H); ^13^C NMR (101 MHz, D_2_O): δ 23.24, 39.53, 56.97, 99.68, 110.18, 121.44 (q, *J* = 269.7 Hz), 122.81, 125.05, 126.55, 129.29, 132.56, 134.16, 136.73, 138.51, 141.22 (q, *J* = 38.4 Hz), 162.27, 171.16, 180.08; LRMS [C_18_H_16_F_3_N_5_NaO_4_] (*m*/*z*): (+ve ion mode) 468.2 [M+Na]^+^.
*(S)-2-Amino-5-(2-(4-oxo-2-(trifluoromethyl)-4,5-dihydropyrazolo[1,5-a]pyrazin-6-yl)acetamido)pentanoic acid IE1775-48* (**1d**), The amide was formed by coupling with (*S*)-methyl 5-amino-2-((*tert*-butoxycarbonyl)amino)pentanoate and the protected product was purified by silica gel chromatography using Hexane:EtOAc:MeOH (4:2:0.5). The final deprotected amide (**1d**) was obtained in 48% yield (over three steps) after purification by silica gel chromatography using EtOAc:MeOH:H_2_O (7:2:1) as a solvent. ^1^H NMR (400 MHz, DMSO-*d*_6_): δ 1.51 (q, *J* = 7.0 Hz, 2H), 1.65 (d, *J* = 7.8 Hz, 2H), 3.06 (s, 3H), 3.20 (s, 4H), 6.94 (s, 1H), 7.53 (s, 1H), 8.37 (s, 1H); ^13^C NMR (101 MHz, DMSO-*d_6_*): δ 25.60, 29.44, 30.79, 31.75, 55.06, 98.89, 106.80, 122.31 (d, *J* = 268.3 Hz), 133.50, 136.66, 139.46 (d, *J* = 37.1 Hz), 161.82, 169.45, 177.64; LRMS [C_14_H_16_F_3_N_5_O_4_] (*m*/*z*): (-ve ion mode) 395.9 [M-H]^−^.

### 4.13. Isothermal Titration Calorimetry

ITC experiments were performed in triplicate on Nano ITC (TA Instruments, New Castle, DE, USA). hFBL and SAM were dissolved in a buffer containing 10 mM HEPES (pH 7.5),150 mM NaCl, 5% DMSO, and 0.5 mM TCEP. The baseline was equilibrated for 900 s before the first injection. 0.5 mM SAM was titrated as 25 injections of 2.0 μL every 200 s, into 100 μM protein with a stirring rate of 150 rpm. The heat change was recorded by injection over time and the binding isotherms were generated as a function of molar ratio of the protein solution. The dissociation constants (Kd) were obtained after fitting the integrated and normalised data to a single site binding model using NanoAnalyze (TA Instruments).

### 4.14. Surface Plasmon Resonance

hFBL was immobilised onto a CM5 series S sensor chip by amine coupling. The protein was diluted in 10 mM sodium acetate buffer pH 5.5 at a concentration of 100 µg/mL and injected onto flow cells 2, 3, and 4 for 420 s at a flow rate of 10 µL/min. The remaining unreacted NHS ester groups were neutralised by an injection of 1 M ethanolamine-HCl (pH 8.0). A total of at least 15,000 RU of fibrillarin was captured per flow cell. Flow cell 1 served as a negative control and underwent the same treatment, without sample injection. This enabled double reference subtraction of the responses (2–1, 3–1, and 4–1). SAM, fragments, and fragment derivatives were flowed over the immobilised proteins at concentrations from 3.2 µM to 2000 µM with 1:5 dilutions in 5% DMSO in HBS-EP+ buffer (0.01 M HEPES pH 7.4, 0.15 M NaCl, 3 mM EDTA, 0.005% *v*/*v* Surfactant P20, 0.5 mM TCEP) at a flow rate of 30 µL/min. SAM was tested both before and after all other fragments, confirming the stability of immobilised hFBL as similar responses were acquired. All affinity calculations were based on triplicate data from three separate flow cells.

## 5. Conclusions

In summary, we have solved the first crystal structure of hFBL in complex with its native cofactor SAM, and have discovered small molecule binders that compete with cofactor binding to hFBL. These structural data are fundamental for future development of hFBL inhibitors that could validate its potential as a therapeutic target.

## Data Availability

Data is contained within the article or supplementary material.

## Supplementary Materials

The following are available online at https://www.mdpi.com/article/10.3390/ph15010026/s1: Figure S1: the hFBL cofactor binding site display high structural similarity to previously reported FBL structures; Figure S2: STD NMR spectra showing binding of 0.5 mM SAM to 20 µM hFBL; Figure S3: root mean square fluctuations (RMSF) of hFBL residues during MD simulations; Figure S4: distribution of similarity scores between each fragment and its closest neighbour; Figure S5: STD NMR analysis of selected fragment hits from 19F NMR detected screening with hFBL; Figure S6: docked poses of top ranking compounds designed to bind at the cofactor binding site of hFBL; Figure S7: STD NMR analysis of selected fragment derivatives with hFBL; Figure S8: representative sensorgrams from SPR analysis of the interaction between hFBL and various ligands; Figure S9: NMR spectra of 1a; Figure S10: NMR spectra of 1b; Figure S11: NMR spectra of 1c; Figure S12: NMR spectra of 1d; Table S1: crystallographic data; Table S2: structural comparison of hFBL with archaeal and fungal fibrillarin homologs; Table S3: binding affinities of compounds with hFBL derived from SPR assays and corresponding docking scores from molecular docking. References [47,64] are cited in the supplementary materials
