# Supplementary material for: Discovery of Cofactor Competitive Inhibitors against the Human Methyltransferase Fibrillarin
Source: Pharmaceuticals (Basel). 2021 Dec 24;15(1):26. doi: 10.3390/ph15010026 (PMC8779173; doi:10.3390/ph15010026)
Supplement: Supplementary file 1 [file pharmaceuticals-15-00026-s001.zip › pharmaceuticals-1493769-supplementary.pdf]

Supporting Information

Discovery of cofactor competitive inhibitors against the human methyltransferase fibrillarin

Yun Shi, Ibrahim M. El-Deeb, Veronika Masic, Lauren Hartley-Tassell, Andrea Maggioni, Mark von Itzstein* and Thomas Ve*

Institute for Glycomics, Griffith University, Southport, QLD 4222, Australia.

Supplementary Figures	Page 2
Supplementary Tables	Page 12
Supplementary References	Page 16

Supplementary Figures

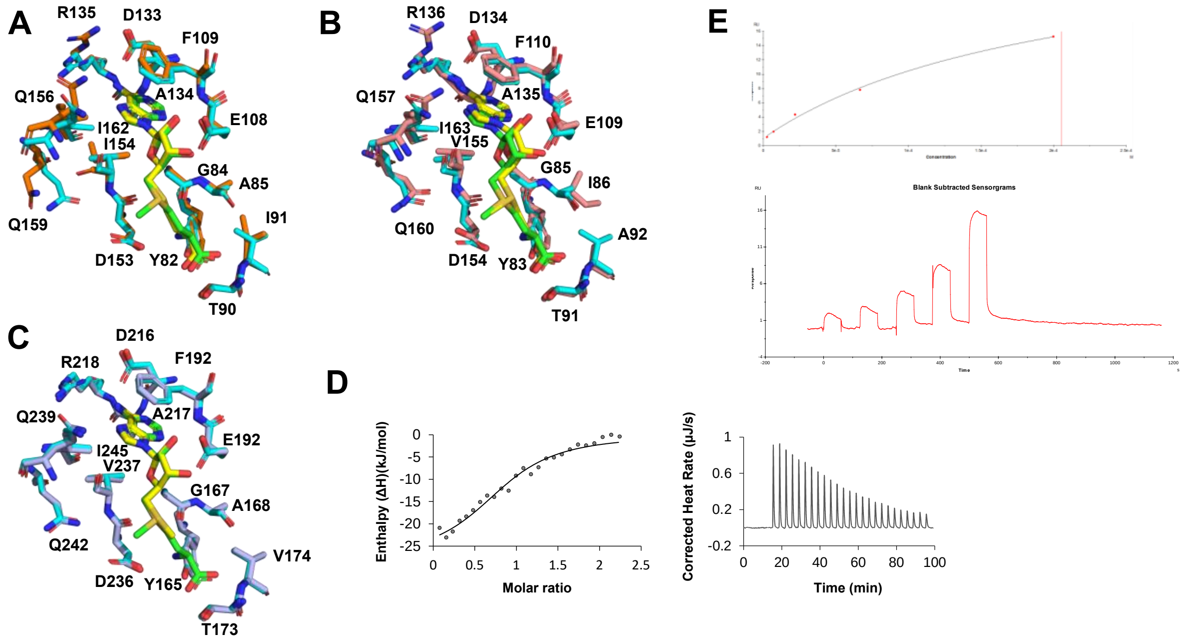

Figure S1. The hFBL cofactor binding site display high structural similarity to previously reported FBL structures. (**A**-**C**) Superimposition (stick representation) of $hFBL^{SAM}$ (residues in cyan; SAM in green) with (A) *Saccharolobus solfataricus* FBL (residues in orange, SAM in yellow), (**B**) *Aeropyrum pernix* FBL^{SAH} (residues in wheat, SAH in yellow) and (**C**) $hFBL^{MTA}$ (residues in light blue, MTA in yellow). The *Saccharolobus solfataricus, Aeropyrum pernix* and human FBL binding site residues are labelled in (**A**), (**B**) and (**C**), respectively. Oxygen, nitrogen, and sulfur atoms are colored in red, blue, and light orange, respectively. (**D**) Integrated (left) and raw (right) ITC data for the titration of 0.5 mM SAM with 100 µM hFBL. (E) SPR sensorgram (bottom) and data fitting (top) for SAM binding affinity with hFBL via SPR.

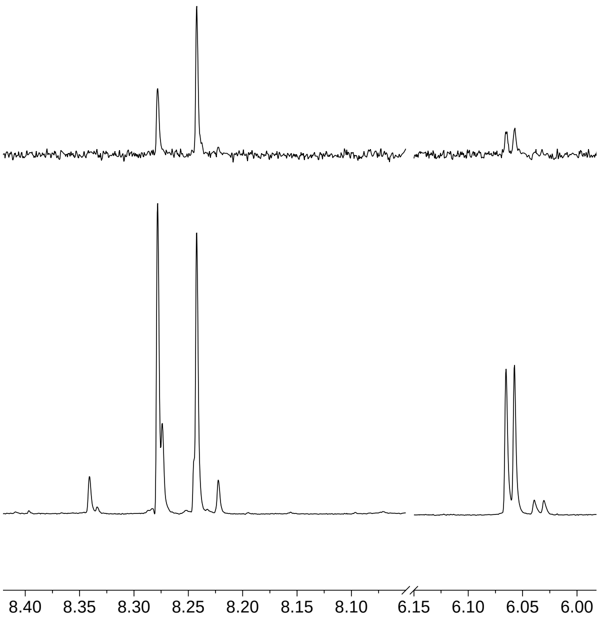

Figure S2. Expansions of reference (bottom) and difference (top, scaled 8X) STD NMR spectra showing binding of 0.5 mM SAM to 20 μM hFBL.

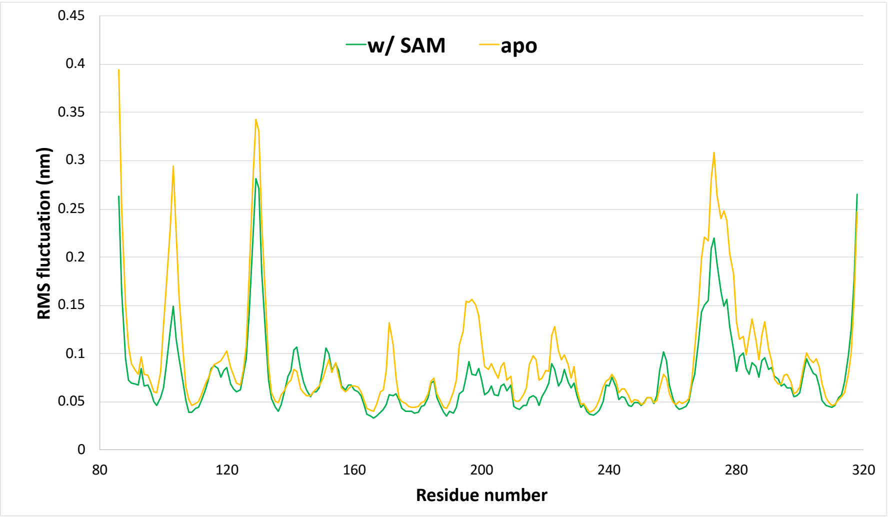

Figure S3. Root mean square fluctuations (RMSF) of hFBL residues during MD simulations. The RMSF values correspond to averages of triplicate MD runs.

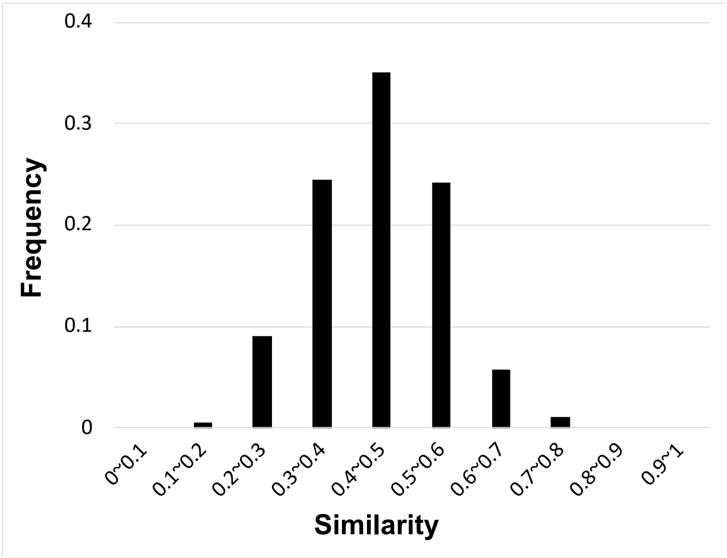

Figure S4. Distribution of similarity scores between each fragment and its closest neighbour.

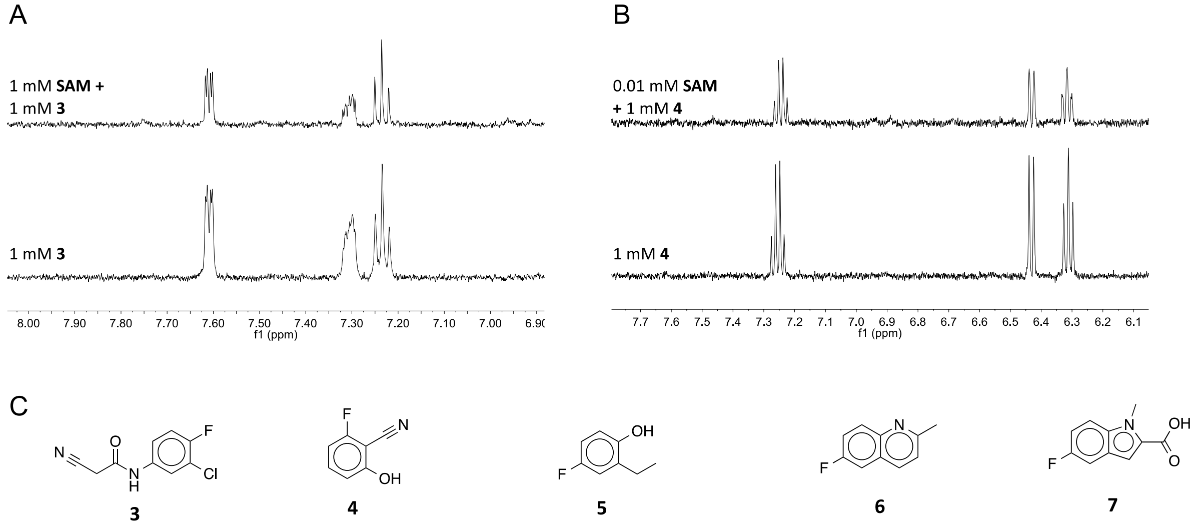

Figure S5. STD NMR analysis of selected fragment hits from ^{19}F NMR detected screening with hFBL. A) Expansions of STD NMR spectra showing the binding of 1 mM **3** to 40 μM hFBL in the absence (bottom expansion) and presence (top expansion) of 1 mM SAM, with 800 scans and 2 sec saturation time. B) Expansions of STD NMR spectra showing the binding of 1 mM **4** to 30 μM hFBL in absence (bottom expansion) and presence (top expansion) of 0.01 mM SAM (bottom expansion), with 1600 scans and 2 sec saturation time. C) Chemical structures of fragment hits identified by ^{19}F NMR detected screening.

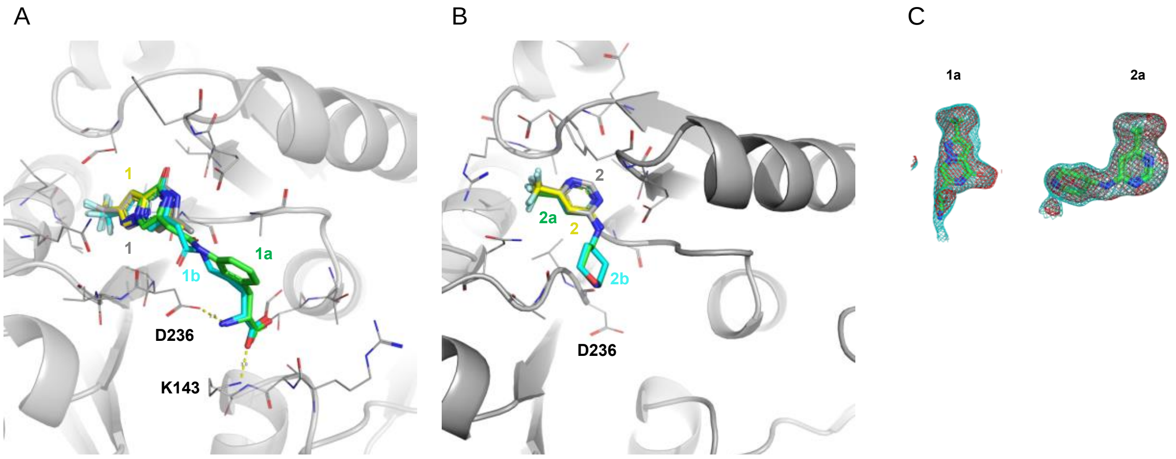

Figure S6. Docked poses of top-ranking compounds designed to bind at the cofactor binding site of hFBL. A) Overlay of docked poses of **1** (yellow), **1a** (green), and **1b** (cyan) with the crystal structure of hFBL in complex with **1** (grey). B) Overlay of docked poses of **2** (yellow), **2a** (green), and **2b** (cyan) with the crystal structure of hFBL in complex with **2** (grey). The protein backbone is displayed in cartoon representation. Ligands and residues are displayed in thick and thin stick representations, respectively. Oxygen, nitrogen and fluorine atoms are coloured in red, blue and pale cyan, respectively. C) Standard omit mFo-DFc (red mesh, contoured at 3.0 σ) and polder (cyan mesh, contoured at 4.0 σ) maps of **1a** and **2a**.

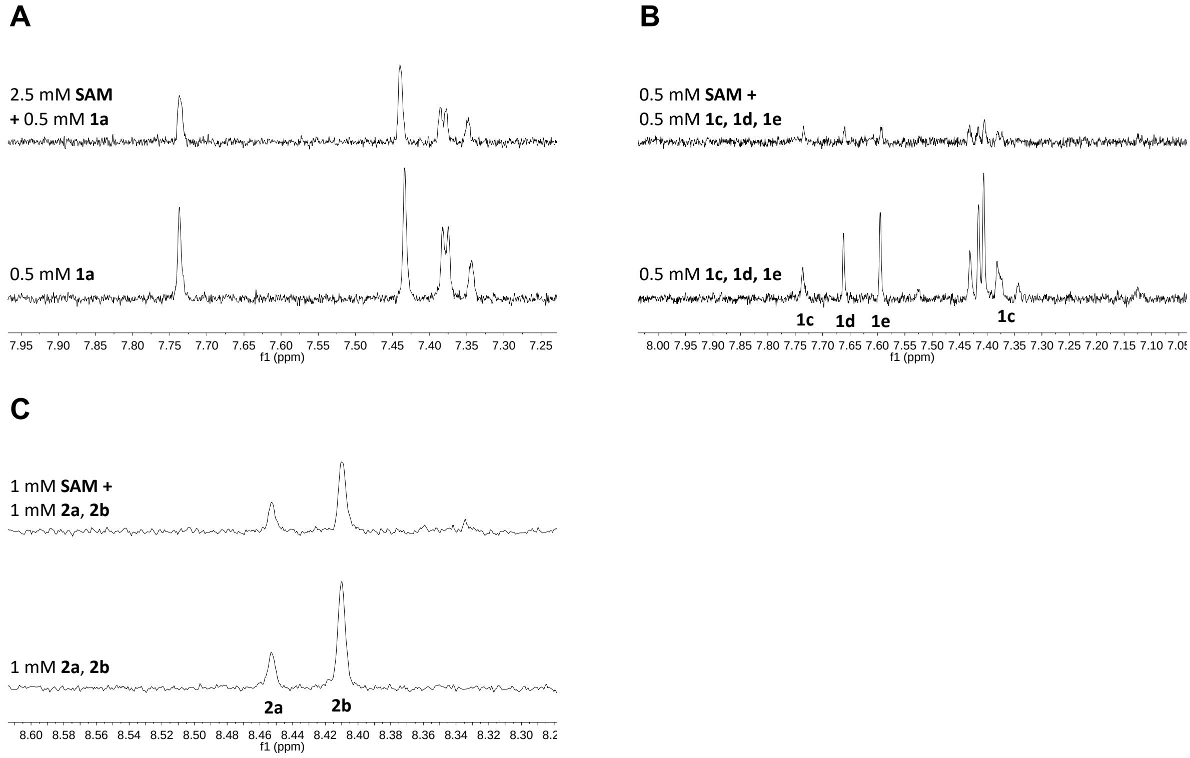

Figure S7. STD NMR analysis of selected fragment derivatives with hFBL in the presence and absence of SAM, respectively. A) STD NMR spectra showing the binding of 0.5 mM **1a** to 20 µM hFBL in the absence (bottom expansion) and presence (top expansion) of 2.5 mM SAM, with 1024 scans and 3 sec saturation time. B) STD NMR spectra showing the binding of 0.5 mM **1c**, **1d**, and **1e** to 20 µM hFBL in the absence (bottom expansion) and presence (top expansion) of 0.5 mM SAM, with 1024 scans and 2 sec saturation time. C) STD NMR spectra showing the binding of 1 mM **2a** and **2b** to 40 µM hFBL in the absence (bottom expansion) and presence (top expansion) of 1 mM SAM, with 512 scans and 3 sec saturation time.

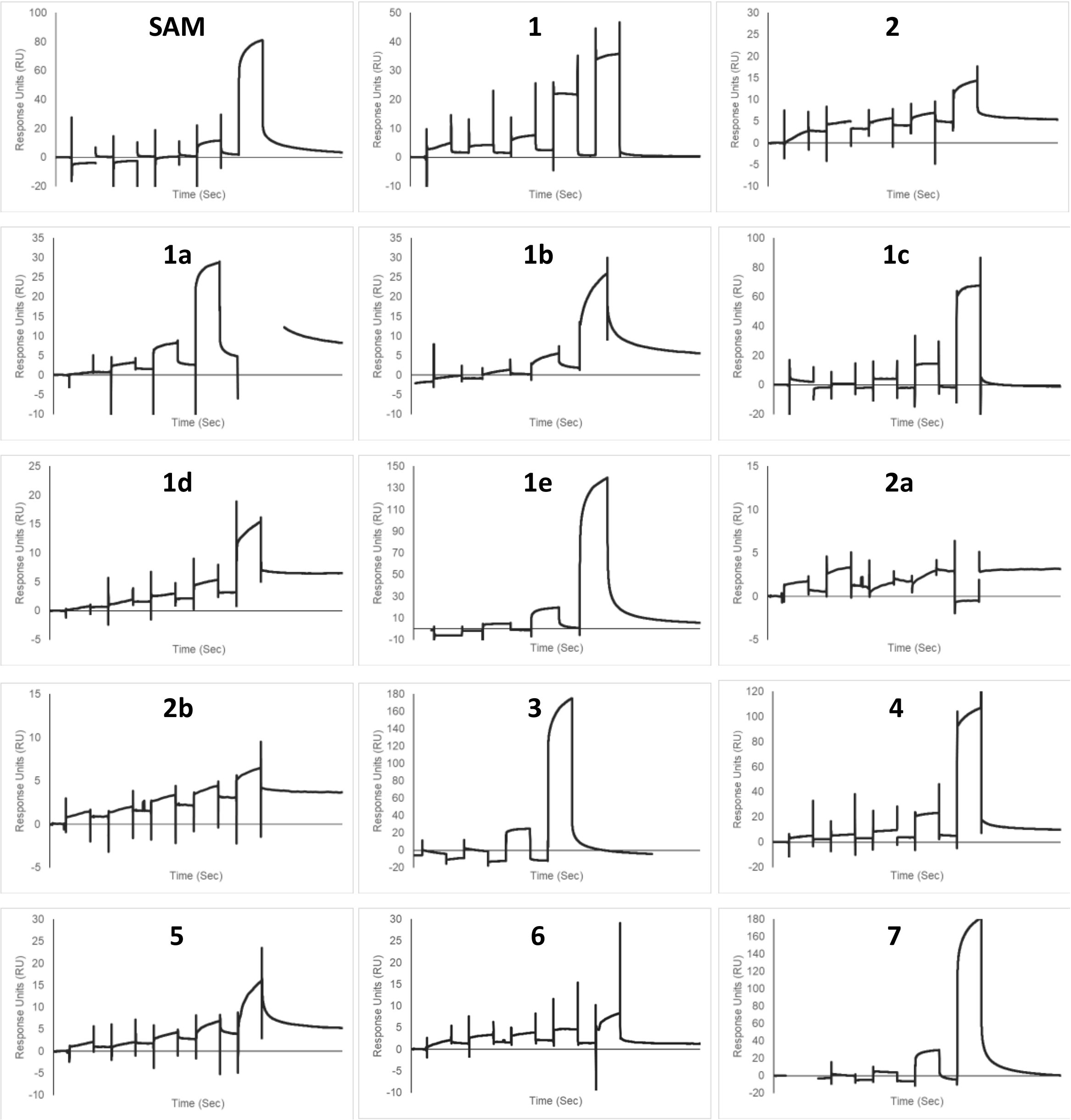

Figure S8. Representative sensorgrams from SPR analysis of the interaction between hFBL and various ligands.

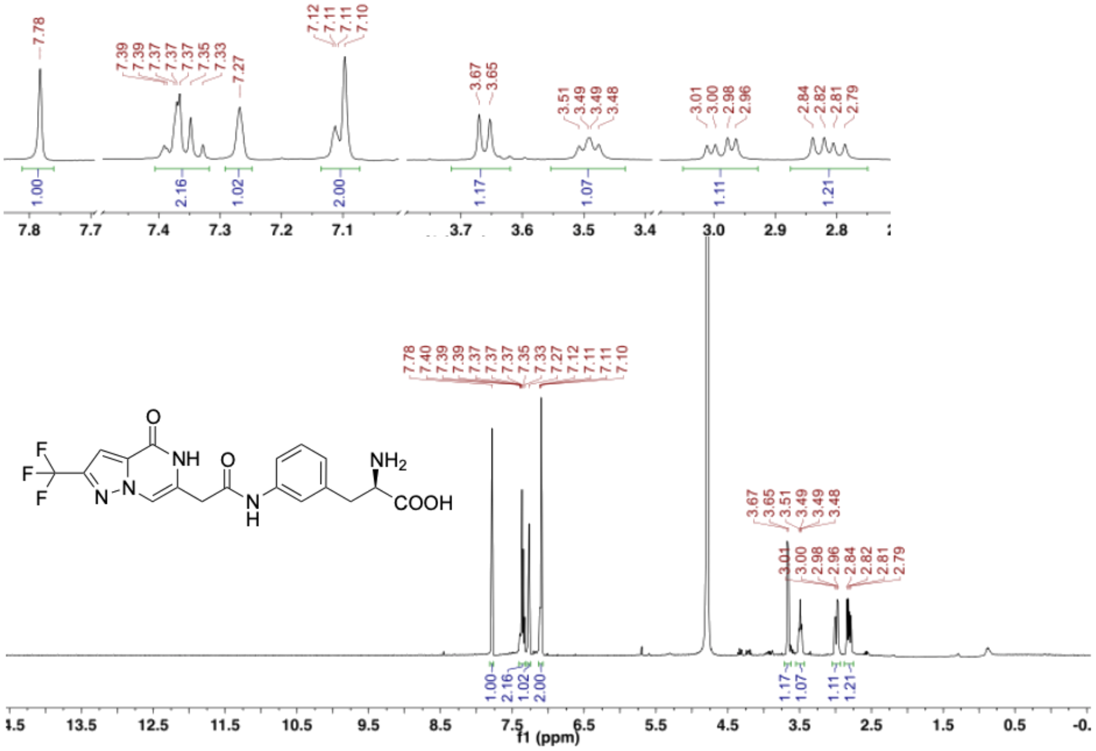

^{1}H NMR Spectrum

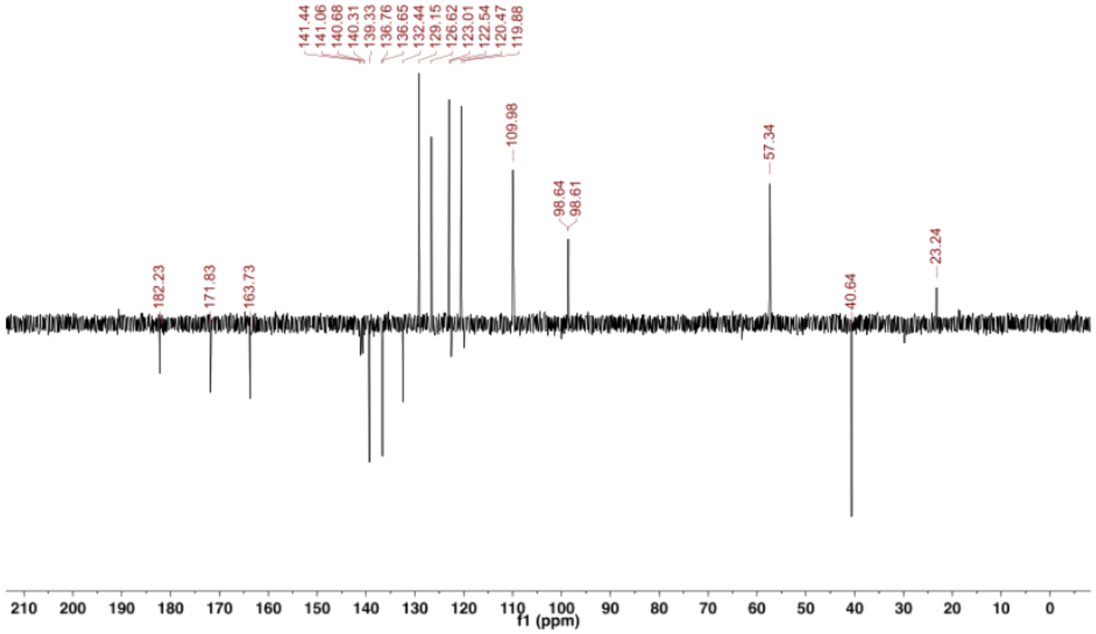

^{13}C NMR Spectrum

Figure S9. NMR spectra of **1a**.

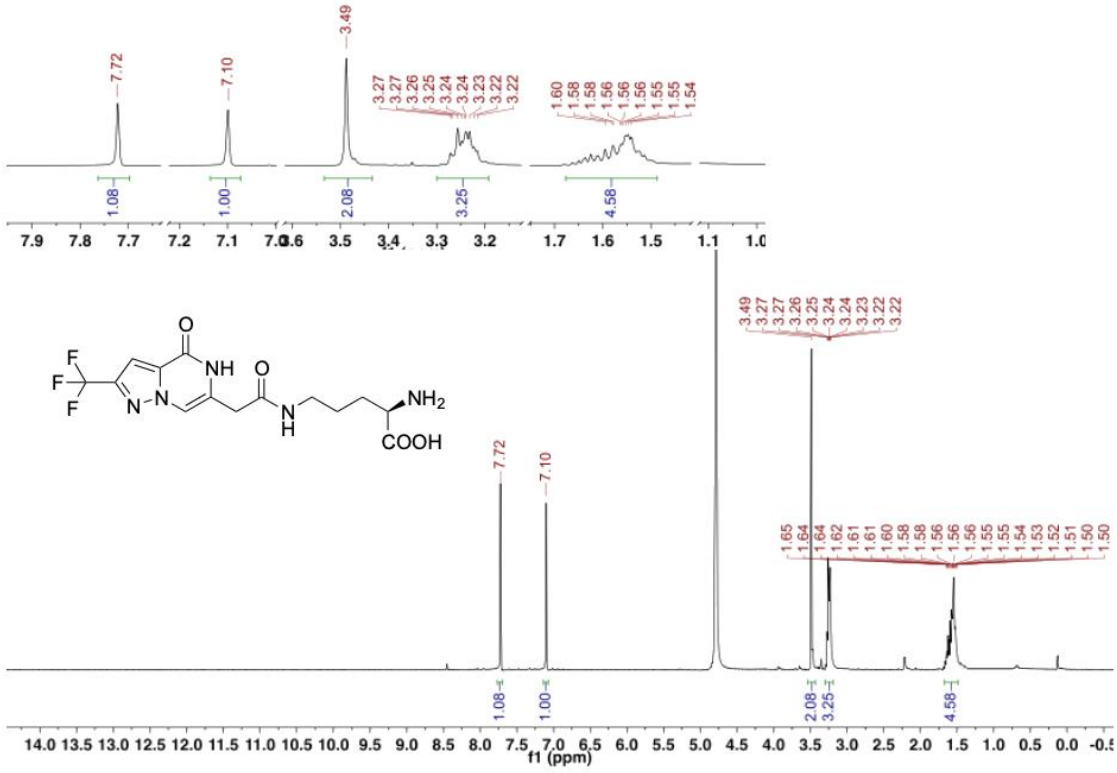

^{1}H NMR Spectrum

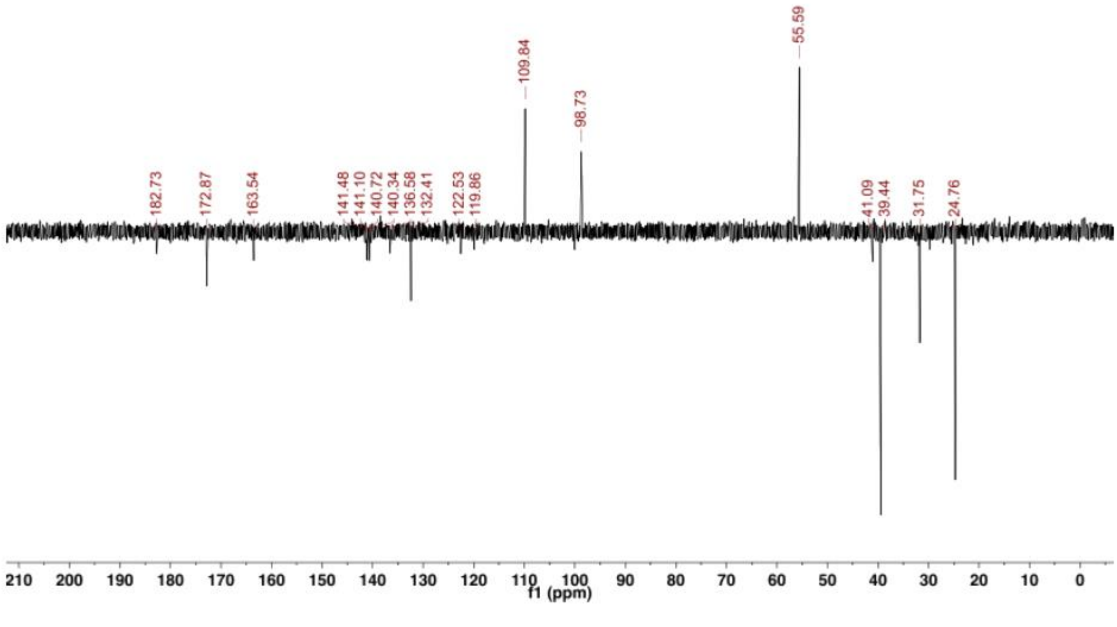

^{13}C NMR Spectrum

Figure S10. NMR spectra of **1b**.

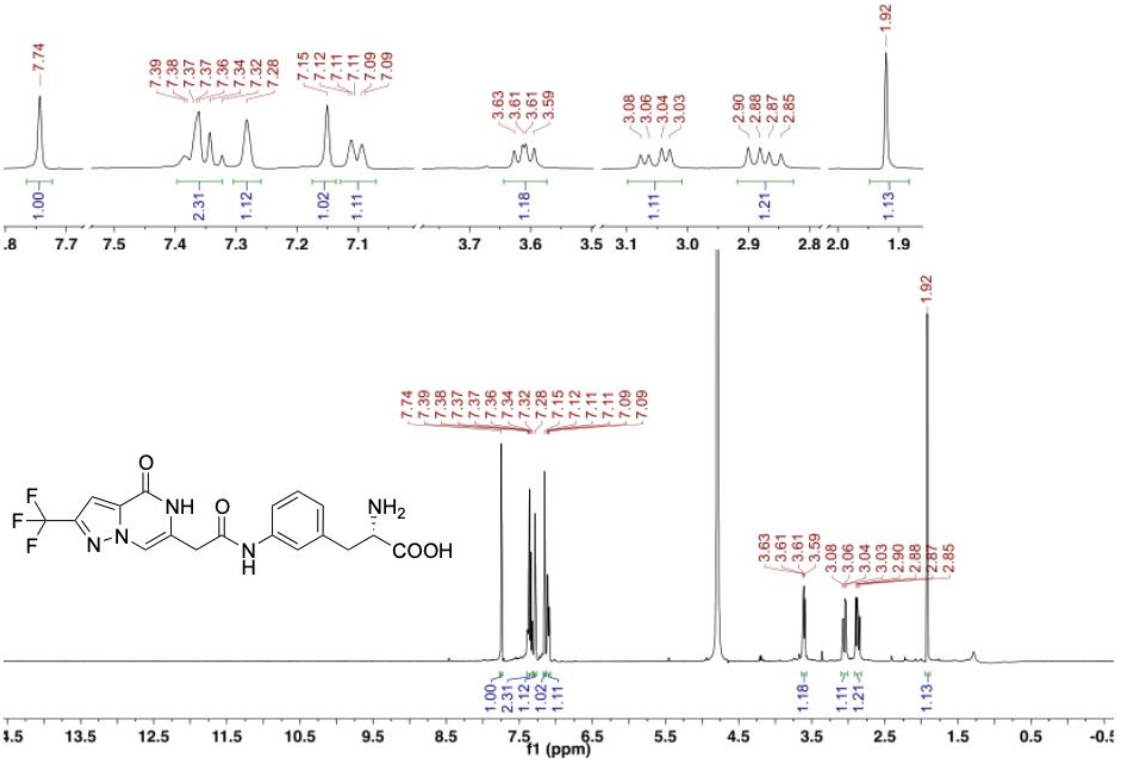

^{1}H NMR Spectrum

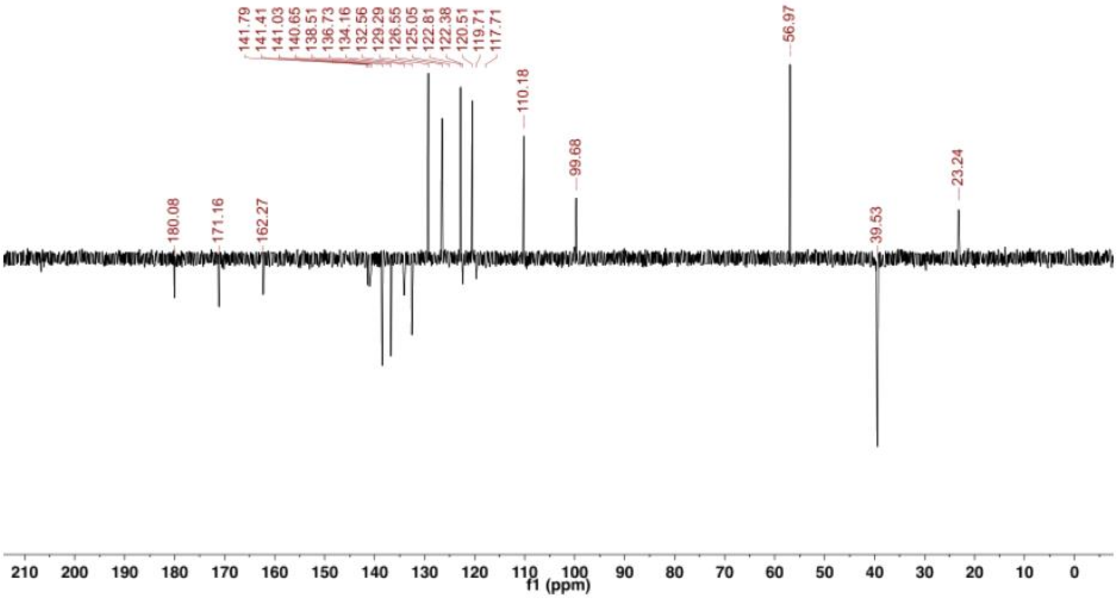

^{13}C NMR Spectrum

Figure S11. NMR spectra of **1c**.

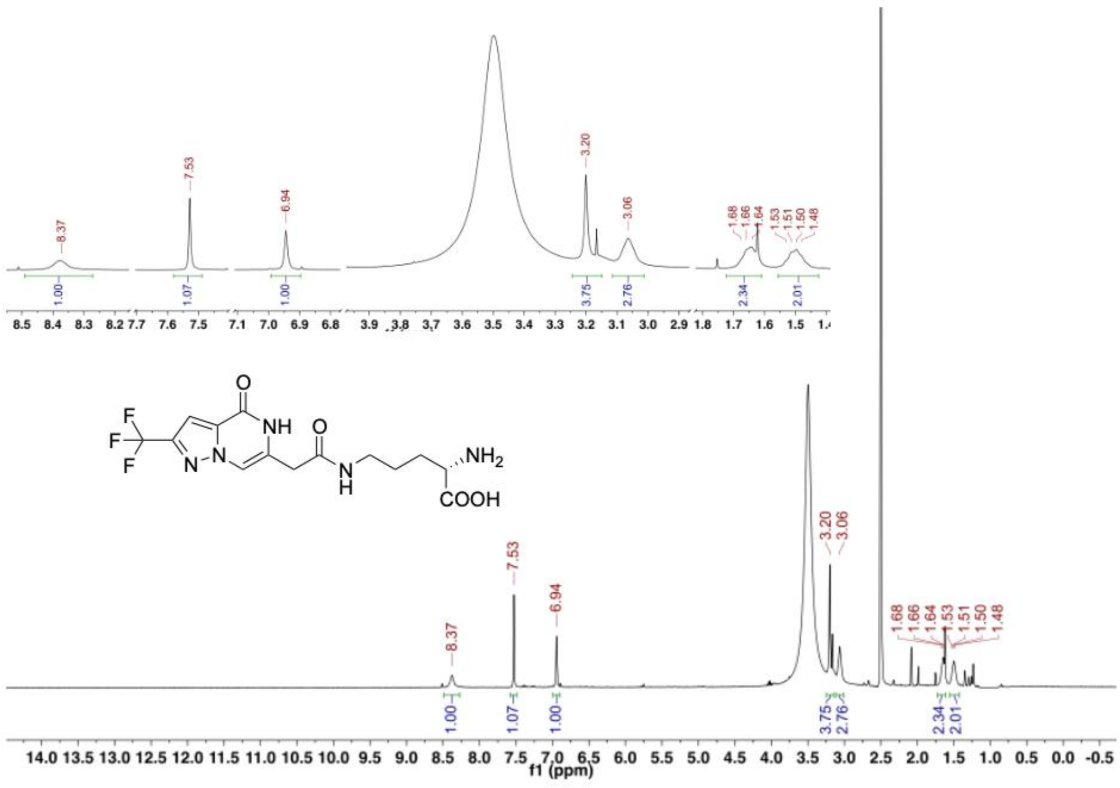

^{1}H NMR Spectrum

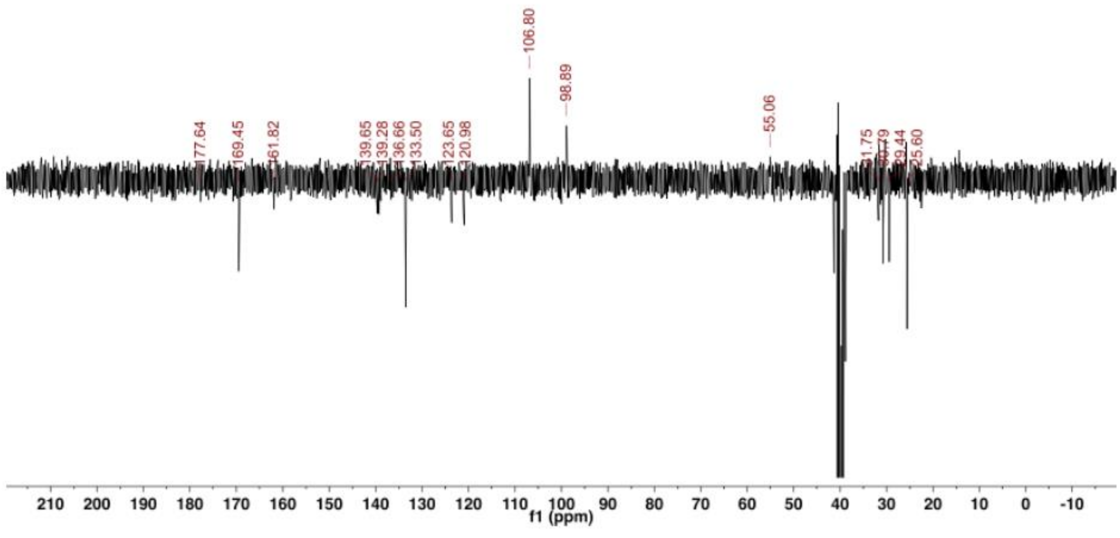

^{13}C NMR Spectrum

Figure S12. NMR spectra of **1d**.

Supplementary Tables

Table S1. Crystallographic data.

	hFBL:Apo	hFBL:SAM	hFBL:1 (cocktail soak)	hFBL:1 (single soak)	hFBL:1a	hFBL:2 (cocktail soak)	hFBL:2 (single soak)	hFBL:2a
**Data collection**								
Space group	C 2 2 21	C 2 2 21	P 21 2 21	P 21 21 2	P 21 2 21	C 2 2 21	C 2 2 21	C 2 2 21
Cell dimensions								
a, b, c (Å)	69.14, 138.19, 66.55	69.28, 138.92, 65.67	68.04, 68.87, 138.65	67.61, 68.97, 138.55	68.38, 68.87, 136.44	69.13, 138.65, 66.43	69.21, 138.90, 66.48	69.29, 139.58, 66.25
α, β, γ (°)	90, 90, 90	90, 90, 90	90, 90, 90	90, 90, 90	90, 90, 90	90, 90, 90	90, 90, 90	90, 90, 90
Resolution (Å)	47.93 - 1.99 (2.04 - 1.99)	47.72 - 1.75 (1.78 - 1.75)	48.40 - 1.75 (1.78 - 1.75)	48.88 - 1.75 (178 - 1.75)	48.53 - 1.90 (1.94 - 1.90)	47.96 - 1.91 (1.95 - 1.91)	48.03 - 1.81 (1.84 -1.81)	48.05 - 1.90 (1.94 - 1.90)
Total reflections	162,232 (11,659)	236,103 (12,290)	486,995 (26,465)	479,341 (24,211)	234,657 (15,320)	182,482 (11,449)	384,489 (16,162)	329,093 (18,603)
Unique reflections	22,206 (1623)	32,353 (1745)	65,541 (3509)	66,058 (3,465)	51,310 (3,233)	25,161 (1639)	29,246 (1,359)	25,684 (1,570)
Completeness (%)	99.9 (99.4)	99.9 (97.9)	98.7 (97.5)	99.7 (96.1)	99.5 (99.3)	99.8 (97.3)	97.5 (77.6)	99.5 (95.9)
Multiplicity	7.3 (7.2)	7.3 (7.0)	7.4 (7.5)	7.3 (7.0)	4.6 (4.7)	7.3 (7.0)	13.1 (11.9)	12.8 (11.8)
Wilson plot B ($Å^2$)	37.0	36.8	18.5	19.0	33.4	24.2	28.4	29.0
Rmeas (%)	5.7 (117.5)	7.8 (77.6)	7.0 (82.2)	5.9 (69.8)	9.0 (127.2)	6.3 (89.9)	8.1 (174.1)	5.0 (47.5)
Rmerge (%)	4.9 (100.7)	6.7 (66.3)	6.0 (71.0)	5.1 (59.8)	7.0(101.1)	5.9 (83.3)	7.5 (159.4)	4.6 (43.5)
Rpim (%)	2.9 (60.2)	4.0- (39.9)	3.5 (41.3)	3.0 (35.7)	5.5 (76.2)	2.3 (33.4)	3.1 (69.0)	1.9 (18.9)
$\langle I/\sigma(I)\rangle$	23.7 (1.7)	13.2 (1.9)	19.8 (2.8)	16.5 (2.3)	7.0 (1.5)	17.4 (2.3)	18.0 (1.2)	22.6 (4.0)
$CC_{1/2}$	1.000 (0.821)	0.999 (0.807)	0.999 (0.822)	0.999 (0.858)	0.999 (0.894)	1.000 (0.865)	0.999 (0.745)	1.000 (0.950)
**Refinement**								
Resolution (Å)	38.33 - 1.99	45.08 - 1.75	48.40 - 1.75	48.88 - 1.75	48.30 - 1.90	45.27 - 1.91	48.03 - 1.81	38.63 - 1.90
Number of reflections used	22171	32329	65454	65967	51057	25138	29200	25, 657
Rwork (%)	22.63	17.89	18.77	18.71	22.25	19.57	19.42	18.86
Rfree (%)	25.95	20.94	21.36	22.26	26.63	22.89	23.28	22.49
Final model								
Number of protein residues	229	229	456	456	452	229	229	228
Number of water molecules	71	189	315	353	115	94	136	89
Number of ligand atoms	3	27	62	62	68	30	20	20
Average B-factor ($Å^2$)								
Protein	51	27	27	27	40	33	36	38
Solvent	46	37	36	37	42	38	43	43
Ligands	FMT: 52	SAM: 19	Compound 1: 39<br>FMT: 40<br>DMSO: 40	Compound 1: 40<br>FMT: 41<br>DMSO:47	Compound 1a: 44<br>FMT: 51<br>DMSO:38	Compound 2: 45<br>FMT: 41<br>DMSO: 32	Compound 2: 47<br>FMT: 47	Compound 2a: 38<br>FMT: 50
R.m.s. deviations								
Bond lengths (Å)	0.013	0.010	0.009	0.010	0.012	0.011	0.011	0.011
Bond angles (°)	1.141	1.047	1.049	1.074	1.131	1.076	1.091	1.105
Ramachandran plot (%)								
Favoured	96.44	97.78	97.77	97.32	97.52	97.33	97.33	98.65
Allowed	3.56	2.22	2.23	2.68	2.48	2.67	2.67	1.35
Outliers	0	0	0	0	0	0	0	0

PDB ID	7SE6	7SE7	7SE8	7SE9	7SEC	7SEA	7SEB	7SED

The statistics were calculated using AIMLESS [1] and MolProbity [2].

Statistics for the highest-resolution shell are shown in parentheses.

$R_{merge} = \sum_{hkl}\sum_{j}|I_{hkl,j} - < I_{hkl} > |/(\sum_{hkl}\sum_{j}I_{hkl,j})$

R_{work} / $R_{free} = \sum_{hkl}\left|F_{hkl}^{obs} - F_{hkl}^{calc}\right|/(\sum_{hkl}F_{hkl}^{obs})$; R_{free} was calculated using randomly chosen 5-10 % fraction of data that was excluded from refinement.

Table S2. Structural comparison of hFBL with Archaeal and fungal Fibrillarin homologs**.**

**Organism**	**PDB ID**	**RMSD**
Methanococcus jannaschii	1G8S	1.3 (231 aligned Cα atoms)
Saccharolobus solfataricus	3PLA	1.0 (228 aligned Cα atoms)
Pyrococcus furiosus	3NMU	1.2 (228 aligned Cα atoms)
Aeropyrum pernix	4DF3	1.5 (231 aligned Cα atoms)
Saccharomyces cerevisiae	6ND4	1.3 (228 aligned Cα atoms)
Chaetomium thermophilum	5JPQ	1.0 (227 aligned Cα atoms)

Table S3. Binding affinities of compounds with hFBL derived from SPR assays and corresponding docking scores from molecular docking.

**Compound**	K_D **(μM)**	**Docking score (kJ mol^{-1})**
SAM	33.7 ± 7.7	
**1**	520 ± 40	-7.74
**1a**	510 ± 50	-8.00
**1b**	6490 ± 720	-7.73
**1c**	2660 ± 40	-7.42
**1d**	3350 ± 480	-7.49
**2**	2700 ± 780	-7.33
**2a**	43.8 ± 2.2	-7.83
**2b**	250 ± 20	-7.59

Supplementary References

1. Evans, P. Scaling and Assessment of Data Quality. *Acta Crystallogr. D Biol. Crystallogr.* **2006**, *62*, 72–82, doi:10.1107/S0907444905036693.
2. Chen, V.B.; Arendall, W.B.; Headd, J.J.; Keedy, D.A.; Immormino, R.M.; Kapral, G.J.; Murray, L.W.; Richardson, J.S.; Richardson, D.C. MolProbity: All-Atom Structure Validation for Macromolecular Crystallography. *Acta Crystallogr. D Biol. Crystallogr.* **2010**, *66*, 12–21, doi:10.1107/S0907444909042073.
